# Structural gender inequalities and symptoms of postpartum depression in 40 countries

**DOI:** 10.1192/j.eurpsy.2022.2208

**Published:** 2022-09-01

**Authors:** P.A. Martinez Diaz, J. Nazif-Munoz, I. Magaña, G. Rojas

**Affiliations:** 1 Université de Sherbrooke, Faculté De Médecine Et Des Sciences De La Santé, Longueuil, Canada; 2 Centre de recherche Charles-Le Moyne sur les innovations en santé, -, Longueuil, Canada; 3 Universidad de Santiago de Chile, Escuela De Psicología, Santiago, Chile; 4 Hospital Clínico Universidad de Chile, Departamento De Psiquiatría Y Salud Mental, Santiago, Chile

**Keywords:** postpartum depression, Gender inequality, Abortion Policies, Meta-regression

## Abstract

**Introduction:**

The extent to which structural gender inequality contributes to macro-level differences in postpartum depression (PPD) remains largely unknown.

**Objectives:**

To examine the association of structural gender inequalities with national-level prevalence estimates of PPD symptoms.

**Methods:**

Meta-analytically derived national-level estimates for the prevalence of PPD symptoms – based on the Edinburgh Postnatal Depression Scale (EPDS) – were combined with economic (e.g., income inequality), health (e.g., infant mortality rate), sociodemographic (e.g., urban population), and structural gender inequality variables (e.g., abortion policies) for 40 countries (276 primary studies). Data came from a prior meta-analysis on PPD prevalence and international agencies (e.g., UNICEF). Meta-regression techniques and traditional p-value based stepwise procedures, complemented with a Bayesian model averaging approach, were used for a robust selection of variables associated with national-level PPD symptom prevalence. Sensitivity analyses excluded primary studies with small sample sizes or countries lacking evidence for psychometric properties of the EPDS.

**Results:**

Income inequality (β = 0.04, 95% CI = 0.02 to 0.07) and abortion policies (β = 0.02, 95% CI = 0.00 to 0.03) were the only variables included in the final, adjusted model, accounting for 60.7% of the variance in PPD symptoms across countries. Gradual liberalizations of abortion policies were associated with a 2% decrease in national-level PPD symptom prevalence. Results were robust to sensitivity analyses.

**Conclusions:**

Structural gender inequalities might be social determinants of PPD, as the liberalization of abortion policies seem to impact women’s perinatal mental health on a population level. More research on structural gender inequality is needed to guide policy and practice.

**Disclosure:**

No significant relationships.

